# Low avascular necrosis rates in femoral neck fractures: efficacy of cannulated screw fixation

**DOI:** 10.1007/s00590-024-03956-0

**Published:** 2024-05-02

**Authors:** S. D. Scattergood, A. L. Berry, O. Flannery, A. Burdon, S. R. Mitchell, J. W. A. Fletcher

**Affiliations:** 1 Department of Trauma and Orthopaedics, Bristol Royal Infirmary, Bristol, UK; 2 Department of Mathematical Sciences, University of Bath, Bath, UK; 3 Department for Health, University of Bath, Bath, UK

**Keywords:** Neck of femur fracture, Hip Fracture, Intracapsular, Cannulated screws, Avascular necrosis

## Abstract

**Purpose:**

Cannulated screw fixation for femoral neck fractures is often limited by concerns of avascular necrosis (AVN) occurring, historically seen in 5–40% of fixed intracapsular fractures. This study aims to assess the outcomes, particularly the AVN rate, associated with current surgical techniques within our unit.

**Methods:**

We conducted a single-center cross-sectional study, manually searching operative records between July 14, 2014, and December 1, 2018, identifying patients with intracapsular fractured neck of femur fixed with cannulated screws, with a minimum of two years follow-up. Patient records and radiographs were reviewed for clinical and radiographic diagnoses of AVN, non-union, post-operative metalwork infection, and screw penetration of the head. Additionally, fracture pattern and displacement, screw configuration, reduction techniques, and adequacy of reduction were recorded, with radiographs independently analyzed by four orthopedic surgeons.

**Results:**

Fifty-six patients were included; average age of 67 years (range 30–100). Forty-two patients (75%) sustained displaced fractures and 14 patients (25%) had undisplaced fractures. Two (4%) patients developed AVN, with no cases of non-union, post-operative metalwork infection or screw penetration of the head. Eight patients (14%) sustained a high-energy injury, though none of these patients developed AVN. All fractures required closed reduction; no open reductions performed. Twenty-seven (64%) of reductions were adequate.

**Conclusion:**

Our observed AVN rate is notably lower than the widely reported figures, even among a significant proportion of displaced fractures that were fixed. This study underscores that with adequate fixation, cannulated screws represent an excellent option for treating intracapsular neck of femur fractures, even in cases of displaced fracture patterns with imperfect reduction.

## Introduction

The treatment approach for intracapsular fractures varies based on fracture characteristics, patient condition, and surgeon preference, with options including fixation or arthroplasty [1]. In elderly, frail, or comorbid patients, arthroplasty is often favored to avoid potential morbidity and mortality associated with a subsequent surgery [2–4]. Conversely, preserving the native joint is prioritized in physiologically healthier patients due to enhanced functionality and concerns regarding the longevity of arthroplasty, particularly in individuals with high activity levels [5–7]. Current UK guidelines recommend arthroplasty for all displaced intracapsular hip fractures, regardless of age or co-morbidities [8]. Undisplaced fractures in the UK are typically treated with either a Sliding Hip Screw (SHS) or multiple parallel cannulated screws (CS), although national guidance does not endorse one over the other due to insufficient evidence [8–11].

In the UK, there is often reluctance to use CS fixation due to perceived high risks of avascular necrosis (AVN) of the femoral head, with reported rates ranging from 5 to 40% [4, 9, 12–14]. Numerous risk factors have been proposed, but their specific impact on adverse outcomes remains unclear, especially with the current multidisciplinary approach to managing hip fracture patients. These factors include patient characteristics, fracture details, adequacy of reduction, and surgical fixation technique [15–17]. This study aims to assess the influence of various surgical and patient-related variables on the development of AVN in intracapsular femoral neck fractures treated with cannulated screws.

## Method

We conducted a single-center retrospective cross-sectional study, ensuring compliance with the STROBE statement guidelines [18]. Electronic operative records between July 14, 2014, and December 1, 2018, were manually searched for patients with an intracapsular femoral neck fracture (FNF) treated exclusively with cannulated screws. Inclusion criteria encompassed patients aged 16 and above who underwent surgical fixation for traumatic FNF between the specified dates. Patients were followed up for a minimum of two years or until death, whichever occurred first. Those with pathological fractures due to malignancy were excluded.

Patient records, both electronic and paper-based, were thoroughly reviewed for documented diagnoses of AVN, non-union, post-operative metalwork infection, and screw penetration of the femoral head, alongside demographic data. Operation notes were scrutinized for surgeon seniority, American Society of Anaesthesiologists physical status classification (ASA), date, time from admission to surgery, reduction method (open or closed), screw diameter, configuration, and use of washers. Follow-up involved clinical reviews and imaging, with telephone contact utilized when in-person visits were hindered by COVID-19 restrictions.

Patients who were alive as of December 1, 2020, who had no documented cognitive impairment and had not had at least their 2 years clinical follow-up, were contacted via telephone by the authors and an oxford hip score completed [19]. If the patient scored below 30 or there was concern that the patient was experiencing symptoms of AVN such as chronic pain, they were invited to attend fracture clinic for assessment and radiographs.

### Categorization of complications

Complications were categorized based on their impact on daily life, distinguishing between minor complications necessitating intermittent analgesia or simple wound management and major complications causing significant pain or mobility issues.

### Radiograph analysis

Pre-operative radiographs and intra-operative fixation image-intensifier (II) films were assessed for degree of fracture displacement, adequacy of reduction, screw configuration, use of washers, and screw penetration of the femoral head. All intra-operative XR assessments were performed using the final fixation images.

Fracture displacement was measured with each surgeon recording the following for both pre-op and intra-op images: Garden [20] and AO classification [21] on the AP film, whether the anterior or posterior cortex was breached and the amount of anterior or posterior tilt on lateral films.

Radiographs were independently analyzed by four orthopedic surgeons. A sample of ten cases (two from each year) were re-analyzed 30 days afterward to measure intra-observer reliability. For this study, the fracture displacement was defined as having two or more of the following on either pre-operative or intra-operative images: Garden 3/4, AO 31B1.3, 31B2.3, posterior and anterior cortex breached, > 10 degrees posterior or anterior tilt on lateral film. In the event of a tie when deciding whether a fracture is displaced or not, the most senior surgeon arbitrated.

Displacement was measured as the percentage of the femoral head located superior and inferior to the neck on the anterior–posterior radiograph, and anterior and posterior on the lateral radiograph. Lateral head/neck angles were recorded. Adequate reduction was defined as < 25% displacement superiorly or inferiorly, < 25% displacement anteriorly or posteriorly and anterior/posterior tilt of < 10 degrees on the lateral.

### Statistical analysis

Statistical analysis involved unpaired, two-tailed t tests for comparing AVN rates, non-union and complication rates between displaced and undisplaced fractures. Regression analysis was performed using backwards stepwise regression to select from the following variables: gender (male, female), Screw diameter (7.3, 7.0 mm), screw configuration (inverted triangle, triangle, rhomboid, other), injury energy (high, low), adequate reduction (yes, no), use of washers (yes, no). Intra- and inter-observational reliability were measured with Fleiss’ kappa and Cohens kappa. Results were considered significant at a family wise error rate of 0.05, and confidence intervals were calculated at 95%. Statistical tests were performed with ‘R’, version 4.0.2 [22].

## Results

**Fig. 1:**
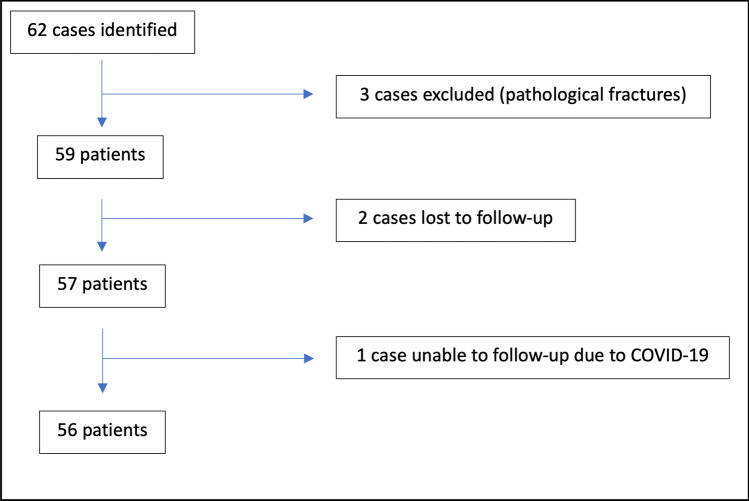
Flowchart of inclusion/exclusion of patients

Fifty-six patients were included in the study (Fig. 1); mean average age 67 years. 71% were female. The majority, comprising 84% of patients, experienced no complications, while minor complications occurred in 12%, and AVN of the femoral head, categorized as a major complication, developed in 4% of patients. Detailed demographic information is presented in Table 1, while Table 2 elucidates risk factors and patient characteristics across complication groups.

**Table 1:** Descriptive data for all patients

Variable	N
*Age*	
Years, mean ± SD	67 ± 18
Min	30
Max	100
*Gender*	
Male	16
Female	40
*Pre-operative mobility*	
Freely mobile without aids (*N*, %)	33
Mobile outdoors with one aid	5
Mobile outdoors with two aids or frame	2
Some indoor mobility but never goes outside without help	16
*Residence before admission*	
Own home	49
Hospital	1
Residential care	4
Nursing home	2
*Length of inpatient rehabilitation*	
Days, mean ± SD	13.4 ± 0.9
Min	1.2
Max	61
*Length of follow-up*	
Months, mean ± SD	33 ± 0.7
Min	24
Max	78
*Oxford hip score*	
Mean ± SD	39 ± 6.7
Min	22
Max	48

**Table 2:** Risk factors compared with all patients, and minor complication group

Risk Factor	None (47)	Minor (7)	All patients (56)
*Age*			
Years, mean	70	62	67
Min	30	37	30
Max	100	73	100
*Residence before admission*			
Own home (*N*, %)	42	6	49
Acute hospital	–	1	1
Residential care	3	–	4
Nursing home	2	–	2
*Pre-fracture mobility*			
Freely mobile without aids (*N*, %)	27	4	33
Mobile outdoors with one aid	3	2	5
Mobile outdoors with two aids or frame	2	–	2
Mobilizes with one stick indoors	–	–	–
Some indoor mobility, never goes outside without help	15	1	16
*Time to surgery*			
Hours, mean	30	25	29
Min	2	9	2
Max	88	42	88
*ASA grade*			
1	7	–	7
2	14	7	23
3	21	–	21
4	5	–	5
5	–	–	–
*Performing Surgeon*			
Consultant	33	4	39
Registrar	14	3	17
*Smoking status*			
Never (*N*, %)	28	3	31
Ex-smoker	12	2	15
Current	7	2	10
*Alcohol intake (per week)*			
Nil	21	3	24
Previous alcohol dependence	3	–	3
0–14 units	18	–	19
15–30 units	2	1	4
31–45 units	2	–	2
> 45 units	1	3	4
*Steroid use*			
None	42	7	51
Intermittent	5	–	5
*Injury velocity*			
High	7	1	8
Low	40	6	48
*Fracture type*			
Undisplaced	11	2	14
Displaced	36	5	42
*Reduction type*			
Closed	36	5	42
Open	–	–	–
*Adequacy of reduction*			
Inadequate	12	2	15
Adequate	24	3	27
*Screw configuration*			
Triangle apex superior	2	–	2
Triangle apex inferior	35	2	33
Rhomboid	14	4	19
Other	1	1	2
*Screw size*		5	
7 mm	25	2	31
7.3 mm	22		25
*Washers*			
Yes	8	–	8
No	39	7	48
*Mobilized on day of surgery*			
Yes	42	6	49
No	5	1	7

Both patients who suffered AVN underwent total hip arthroplasty, constituting the only instances of re-operation. Table 3 delineates the characteristics of these patients. Notably, there were no reported cases of non-union, post-operative metalwork infection, or screw penetration of the femoral head. Additionally, 7% of patients reported prominent metalwork or mild discomfort. Eight patients (14%) sustained a high-energy injury, though none of these patients developed AVN. Forty-two patients (75%) sustained a displaced fracture and 14 patients (25%) had undisplaced fractures. Two patients had no pre-operative lateral radiograph, so interpretation was based solely on intra-operative radiographs. All reductions were performed closed, and 27 of the 42 displaced fractures (64%) were reduced adequately. Mal-reduced fractures had a complication rate of 20% and reduced fractures 11% (*P* = 0.430).

**Table 3:** Two patients had major complications (AVN of the femoral head)

Variable	Patient 1	Patient 2
Date of Surgery	20/9/2014	4/7/2018
Age (years)	78	42
Sex	Female	Female
Residence before admission	Own home	Residential care
Pre-fracture mobility	Freely mobile without aid	Freely mobile without aid
Time to surgery (hours)	49	16
ASA grade	2	2
Performing surgeon	Consultant	Consultant
Length of inpatient rehabilitation (days)	3.6	5.4
Smoking status	Ex-smoker	Current
Alcohol intake (per week)	0–14 units	15–30 units
Steroid use	None	None
Injury Velocity	low	low
Fracture type	Undisplaced	Displaced
Reduction type	Closed	Closed
Adequacy of reduction	Adequate	Inadequate
Screw configuration	Triangle apex inferior	Rhomboid
Screw size	7.3mm	7mm
Washers	No	No
Length of follow-up (months)	68.4	24
Mobilized on day of surgery	Yes	No

Inter-observer reliability was assessed using Fleiss’ kappa value (0.583), and intra-observer reliability with Cohens’ kappa (0.526) demonstrating strong moderate agreements.

The most used screw construct was an inverted triangle (59%), with four screws in a rhomboid form being used in 34% of cases. Screw diameter of 7 or 7.3 mm was used equally (31 vs. 25) and washers were employed in 8 cases.

Median time from admission to surgery was 25 h, all operations were performed in normal working hours on a dedicated orthopedic trauma list, whether weekday or weekend and in 70% of cases, the operation was performed by a consultant surgeon. All of our patients were encouraged to mobilize on the day of surgery, and 88% achieved this goal.

Twenty-eight patients had telephone follow-up as a surrogate for clinical review due to COVID-19. The average oxford hip score was 39 (95% CI 36.5–41.5). One patient scored 22 and was called to clinic for radiographic assessment, which showed union and no AVN. One patient scored 27 and was invited to attend clinic but declined due to risk concerning COVID-19.

At the time of writing, 18 patients had died: 12 patients before their 2-year follow-up was completed and six after 2 years. These patients were included as not having AVN based on an intention-to-treat analysis as they had no documented signs of symptoms of AVN at the time of their death, in keeping with other similar studies into AVN [23].

Multivariate linear regression analysis revealed no correlation between AVN risk or reoperation and displacement, gender, screw configuration, size diameter, or washer usage (*p* ≥ 0.999).

## Discussion

With appropriate fixation, complications including AVN were infrequent. Indeed, our observed rate is lower than recorded elsewhere [4, 9, 12–14, 24]. For comparison, Suarez et al. recently found an overall complication rate with Hemiarthroplasty of 21.7% between 2010 and 2017 through the NSQIP database [25]. Our low rate of AVN has been achieved in a cohort of patients with an average age of 67, 45% current or ex-smokers, 23% excess alcohol use and 75% displaced fractures. All of these factors are thought to increase the risk of poor outcomes including AVN [16, 17].

Our study lacks the statistical power to correlate the causes of AVN due to the low observed rate. However, it is noteworthy that only 7% of patients reported mild pain or discomfort related to prominent metalwork, even though none required screw removal. Telephone follow-up revealed an average Oxford Hip Score of 39, indicating excellent outcomes post-fixation.

All reductions were performed closed on a traction table in the operating room under II guidance, with 64% reduced adequately. With stable fixation, even our mal-reduced fractures’ rate of complications is low (20%). In addition, closed reduction avoids the increased infection risk from open surgery and therefore usually open reduction is not indicated. The most common constructs used at our center were inverted triangle (59%) or rhomboid (34%), which reflects current literature [5, 26–28].

Current guidance from NICE regarding hip fractures [8], recommends that all displaced intracapsular hip fractures regardless of age or co-morbidities should be treated with arthroplasty. This is based on a Norwegian trial from Frihagen et al. [12] in 2007 which found improved functional results with bipolar hemiarthroplasty against fixation using two cannulated screws in 222 patients. An evidence update in 2013 outlines Bjornelv et al.’s [29] paper from which NICE draws the conclusion that arthroplasty is superior to fixation for treatment of all displaced intracapsular fractures in terms of both patient outcomes and cost-effectiveness. We argue that the femoral head should be preserved where possible in order to harvest benefits such as longevity, better functional outcome and lower complication rates than arthroplasty for trauma [5, 25]. One common issue when comparing treatment regime for intracapsular hip fractures is how displacement is viewed, highlighting how the Garden classification may under classify displacement; perhaps to be expected as it only classifies coronal displacement. This is highlighted with a recent paper from Sluliitel et al. [24] that reviewed undisplaced fractures (Garden 1 and 2), though 21% of these had a posterior tilt ≥ 20 degrees. We feel our findings show that focusing on the displacement is not the main priority in that it is the stability of the fixation that is key, rather than the displacement and reduction.

Bjornelv et al. published a cost-utility analysis in 2012 based on data from the RCT by Frihagen in 2007 comparing hemiarthroplasty to internal fixation for displaced intracapsular fractures in 166 patients with an average age of 82, almost half of which were medically co-morbid [12, 30]. Patients were excluded from the trial if they were unfit for arthroplasty, but no patients were judged to be unfit for fixation. The implants compared are not commonly used in the UK: charnley-hastings bipolar (DePuy Synthes) for arthroplasty and two cannulated screws (Olmed, DePuy Synthes) for fixation. These factors favored their finding of hemiarthroplasty superiority from the outset. They also showed an increase of 0.15 quality-adjusted life years (QALYs) (*p* = 0.02) for patients undergoing hemiarthroplasty. Costing analysis showed a trend toward fixation being more expensive in total, but this was not statistically significant [30]. Furthermore, cost for the initial inpatient stay was lower for the fixation group, and the higher total expense was due to their high re-operation rate of 52% which was included in overall costs [30]. Our observed re-operation rate was 3.6% which is lower than 17.9% and 22% documented by Ramadanov and the Fixation using Alternative Implants for Treatment of Hip fractures (FAITH) study respectively [9, 16]. Regardless, a 22% re-operation rate is less than half of what Bjornelv observed and therefore it is likely that in fact, fixation is the more cost-effective option. This would need to be the focus of further research.

A 2011 Cochrane review by Parker et al. concluded no significant difference in fracture healing outcomes for SHS versus CS [10]. The FAITH study found no statistical difference in re-operations at 24 months between implants but the AVN rate was significantly higher in the SHS group (9% vs. 5%)[9].

Fixation with cannulated screws (CS) in an inverse triangle shape with three screws or four screws in a rhomboid formation are optimum configurations, but it is still unclear if either configuration is superior to the others [5, 15, 26, 28, 31, 32]. Guo et al. [27] showed no difference in outcomes between three and four screw constructs, but advised that increased expertise in placing four screws and better guidance around the specific construct shape would help. The authors felt that four screws applied in a four quadrant parallel peripheral (FQPP) construct as shown by Satish et al., with the screws placed as parallel and peripherally in the neck as possible is the best construct, and four screw fixation has been shown to superior if posterior comminution is present [5, 32]. It is known that decreased spread of screws in any construct increases failure rate [28, 33].

Our operative technique differs slightly from the above techniques. Regardless of construct, our first screw was passed across the least comminuted aspect of the fracture. This enables good sub-cortical purchase in the femoral head and allows compression across the fracture site which promotes primary bone healing [27]. Subsequent screws were placed, with the most comminuted aspect of the fracture fixed last. These are intracapsular not intra-articular fractures, and the focus should be on perfect fixation not perfect reduction [5].

Strengths of our study include a matched demographic of patients from the same trauma unit, consistent treatment protocols, and surgery performed by the same group of surgeons over a 4-year period with the only difference being configuration of screws used.

### Limitations of this study

Our study is retrospective in nature and our relatively small sample size is in keeping with other available studies. We acknowledge possible collection bias due to multiple codes used for cannulated screw fixation on our digital surgery tracking software.

## Conclusion

In conclusion, our study underscores that closed, adequate reduction and stable fixation yield a low complication rate, making fixation with CS a safe and reliable option for intracapsular fractures, offering benefits over hemiarthroplasty, such as lower complication rates and improved functionality through femoral head preservation.
